# Immunomodulatory effects of black solo garlic (*Allium sativum* L.) on streptozotocin-induced diabetes in Wistar rats

**DOI:** 10.1016/j.heliyon.2021.e08493

**Published:** 2021-11-27

**Authors:** Saryono, Desiyani Nani, Atikah Proverawati, Sarmoko

**Affiliations:** a Nursing Department, Faculty of Health Sciences, University of Jenderal Soedirman, Dr. Soeparno Street Purwokerto 53123, Indonesia; b Nutrition Department, Faculty of Health Sciences, University of Jenderal Soedirman, Dr. Soeparno Street Purwokerto 53123, Indonesia; c Pharmacy Department, Faculty of Health Sciences, University of Jenderal Soedirman, Dr. Soeparno Street Purwokerto 53123, Indonesia

**Keywords:** *Allium sativum*, Black solo garlic, Immunomodulator, Interferon, Interleukin, Streptozotocin-induced diabetes

## Abstract

Diabetes mellitus is a chronic disease that leads to different complications. Therefore, this study aims to investigate the immunomodulatory effects of the black solo garlic on streptozotocin (STZ)-induced diabetic rats. The Wistar rats were grouped into six groups of: normal control, negative control, treatment dose of 6.5 g/kg, 13.5 g/kg, and 26 g/kg body weight, and positive control glibenclamide. In addition to normal control, rats were induced with STZ on day 8–11. Also, steeping black solo garlic or glibenclamide was administered on the day 12–19. The experimental animals were sacrificed on day 20 and tumor necrosis factor (TNF-α), interleukin-6 (IL-6), interleukin-1β (IL-1β) and interferon gamma (IFN-γ) were measured using ELISA. The results showed that the administration of steeping black solo garlic significantly decreased levels of IL-1β, IL-6 and TNF-α as well as increased IFN-γ with the immunity of STZ-induced rats.

## Teaser

Black solo garlic; Immunomodulator; Interleukin; Interferon.

## 1 Introduction

Diabetes mellitus (DM) is a disease characterized by impaired metabolism of carbohydrates, proteins and fats due to insufficient secretion of insulin. Furthermore, this complication is caused by decreased insulin sensitivity in the tissues, and it is the leading cause of kidney disease, blindness, and amputations [1]. Diabetes mellitus is one of chronic inflammatory disease that suppresses cellular immunity in diabetic [2].

Diabetes mellitus is a chronic condition that occurs when the pancreas do not create enough insulin, or when the body cannot use the insulin adequately. Insulin deficiency in diabetes increases blood glucose resulting in impaired microcirculation as well as increased oxidative stress resulting in prolonged inflammation [3]. This condition is different from inflammation in general. Therefore, agent that can lower blood glucose as well as an anti-inflammatory is required. Diabetes mellitus is associated with high levels of cytokines TNF-α, IL-6, IL-1β, and decreased interferon-γ [4, 5]. TNF-α may change permeability of gromerular and albuminaria by promoting local reactive oxygen species (ROS) production [6]. IL-6 is a powerful predictor of the development of diabetes complications such as diabetic nephropathy [7].

The body's immunity can increase through nutritious food, adequate rest, and routine exercise. In addition, some plants have beneficial effect as immunomodulator such as garlic (*Allium sativum* L.). Fermentation of garlic produced a black garlic with a sour taste rather than a pungent flavor [8]. Black garlic contains reduced sugars, polyphenols, flavonoids, Amadori and Heyns, leucine, isoleucine, phenylalanine, *S*-allyl-cysteine, and alkaloids content higher than those of fresh garlic [9]. Black garlic have several functions, such as an antioxidants, anti-bacterial, anti-allergic, anti-diabetic, anti-inflammatory, and anti-carcinogenic effects [10]. Black garlic increases natural killer cell cytotoxicity and increases the proliferation of B and T lymphocytes and macrophages. The previous study showed that black ordinary garlic extract increases the immune system [11] and recover kidney cells [12].

One of type garlic is single clove or solo garlic (*Allium sativum* ‘Solo garlic’), which contains unstable compounds such as allicin [13]. The allicin ratio in a single clove is equivalent to 5–6 of ordinary garlic. In addition, black solo garlic (BSG) contains more *S*-allyl-cysteine six time than fresh garlic, which increase antioxidant activity [14]. Furthermore, the content of polyphenols, vitamins, minerals and flavonoids are increased during the fermentation [8]. However, there has not been any study on immunomodulatory as well as an anti-inflammatory of black solo garlic. Therefore, this study aims to evaluate the immunomodulatory and anti-inflammatory effect of black solo garlic in streptozotocin (STZ)-induced rats. The present study was designed to investigate the changes of proinflammatory mediators (TNF-α, IL-6, IL-1β, IFN-γ) to verify the protective effects of BSG in DM rats.

## 2 Materials and methods

### 2.1 Plant

The fresh solo garlics were obtained from the Brebes region in Central Java and validated by the Plant Taxonomy Laboratory at the Faculty of Biology at Jenderal Soedirman University, Indonesia.

### 2.2 Fermentation of solo garlics

The selected garlics were approximately the same size and were fermented in the fermentation modified apparatus by using rice cooker. Furthermore, they were arranged in different layers and covered with tissue paper. The rice cooker was set in the warm mode (temperature 60–80 °C) left for 21 days and discoloration was observed once every 3 days. After 21 days, the garlic turned into a black chewy texture.

### 2.3 Preparation of aqueous black solo garlic extract

Black solo garlic were peeled and weighed according to the dose, then mashed using a pestle and mortar. The crushed garlic was placed in a glass, then hot water (at a temperature of 80–90 °C) was added and stirred for 15 min. The mixture was filtered to obtain steeping black solo garlic. The steeping were further analysed by using GC-MS, and we found the same results as reported by Tran et al. (2018) [15].

### 2.4 Animals

The study was conducted using a pre- and post-test approach with a control group design after obtaining ethical eligibility from ethics committee, Faculty of Health Sciences, Jenderal Soedirman University No: 152/EC/KEPK/VII/2020. White male Wistar strain rats aged around 6–7 weeks with a body weight range of 150–200 g were used. They were given *ad libitum* access to standard food and distilled water.

### 2.5 Streptozotocin-induced diabetic rats

Streptozotocin was dissolved in 2.5 ml of citrate buffer 0.05 M and the experimental animals were induced by STZ 50 mg/kg body weight on the day 8th. In addition, they were fasted for 6–8 h before STZ induction. Induction was performed for 3 days.

### 2.6 Treatments

**Figure 1:**
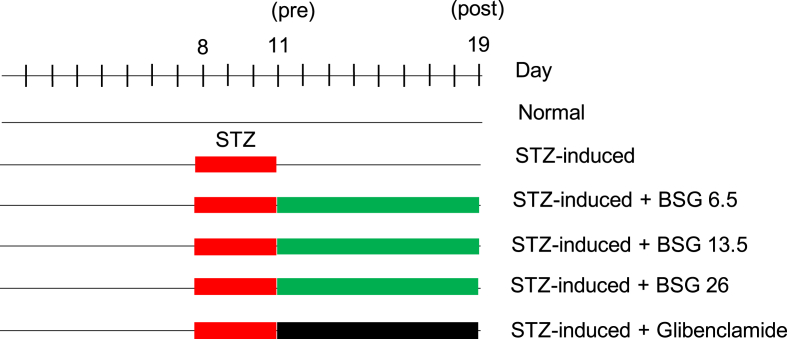
Study design in animal experiments. STZ, streptozotocin; BSG, black solo garlic.

The total number of animals was 30 rats, which were divided randomly into six group: (i) normal, (ii) STZ-induced rat, (iii) STZ-induced rat treated with glibenclamide 0.09 mg/kg body weight (positive control), STZ-induced rat treated with steeping black solo garlic dose of (iv) 6.5 (low dose), (v) 13.5 (medium dose), and (vi) 26 g/kg body weight (high dose). After STZ-induction for 3 days, the animals were treated with steeping black solo garlic for 7 days on the day 11 to 19^th.^ The same schedule was also applied for glibenclamide treatment (Figure 1). Briefly, the black solo garlic powder was measured based on the bodyweight of rats to obtain those three doses. The measured powder was diluted in the 3.6 mL hot water and stirred for 15 min to obtain steeping of black solo garlic. Steeping black solo garlic was given per oral once at night based on the dose and body weight of each group.

### 2.7 Measurement of cytokines

Blood was drawn using a hematocrit capillary pipette which was inserted into the *plexus orbitalis* at the corner of the eye in the day 11^st^ and 19^th^. The ELISA kits were used to check cytokine IL-6, TNF-α, IL-1β, and IFN-γ (BT Laboratories, Shanghai) based on the manufacturer protocol. Briefly, this ELISA kit uses the Sandwich-ELISA principle. The plate has been pre-coated with an antibody specific to rat cytokine. The optical density (OD) was measured using the ELISA Reader (Labotrone, Germany) at a wavelength of 450 nm. The OD value was proportional to the concentration of rat cytokine. Concentration of rat cytokine in the samples was calculated by comparing the OD of the samples to the standard curve.

### 2.8 Statistical analysis

All data were presented as mean ± SEM. GraphPad Prism 8 (California, US) was used to generate the figure. Furthermore, the mean scores among the treatment groups were compared with the one-way ANOVA with a Tukey post-hoc test. Pre- and post-test treatment were analyzed by t-test. p < 0.05 was considered as significant.

## 3 Results

### 3.1 The effect of steeping black solo garlic on pro-inflammatory cytokines

**Figure 2:**
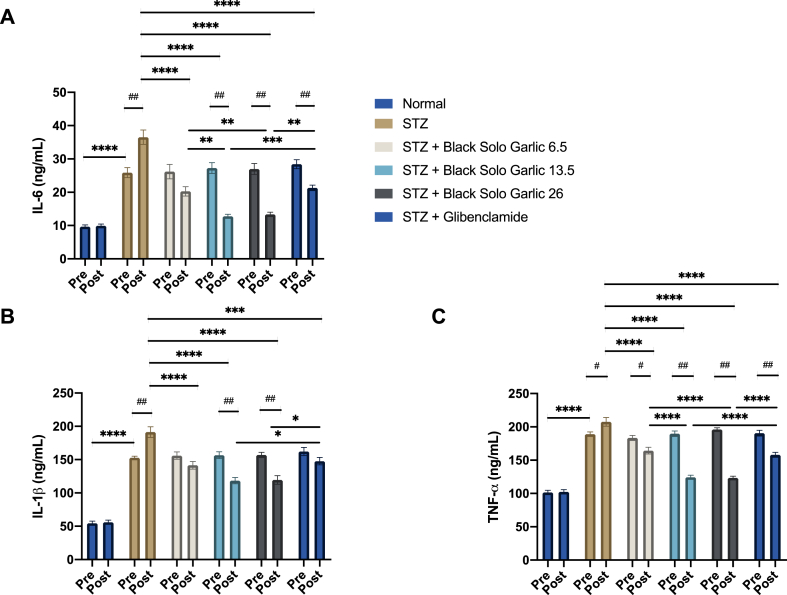
Treatment of black solo garlic reduced pro-inflammatory cytokines. (A) IL-6, (B) IL-1β, (C) TNF-α level in the treatment of black solo garlic in 6.5, 13.5, 26 g/kg doses and glibenclamide 0.09 mg/kg in streptozotocin-induced rats were determined by ELISA. Statistical significance for the difference among groups: ∗∗∗∗p < 0.0001; ∗∗∗p < 0.0005; ∗p < 0.05. Statistical significance for the difference between the data of pre-test group vs post-test groups: ^##^p < 0.01; ^#^p < 0.05.

The results showed that STZ induction significantly increased IL-1β, IL-6, and TNF-α level compared normal group (p < 0.0001) (Figure 2). The administration of black solo garlic at dose of 6.5 g/kg, 13.5 g/kg and 26 g/kg body weight had significantly reduced the level of IL-1β, IL-6, and TNF-α compared to untreated diabetic rats (p < 0.0001). Interestingly, the treatment of black solo garlic at dose of 13.5 g/kg and 26 g/kg body weight was able to reduce IL-1β, IL-6, and TNF-α level lower than those of glibenclamide. The effects on IL-1β, IL-6, and TNF-α were dose dependent, the reduced level of those cytokines in rats administrated with the medium and high black solo garlic dose (13.5 and 26 g/kg, respectively) being significantly greater than that in the rats receiving low black solo garlic dose (6.5 g/kg). Therefore, treatment of the black solo garlic dose of 13.5 g/kg are equal to those 26 g/kg body weight as an anti-inflammatory agent.

### 3.2 The effect of steeping black solo garlic on IFN-γ

**Figure 3:**
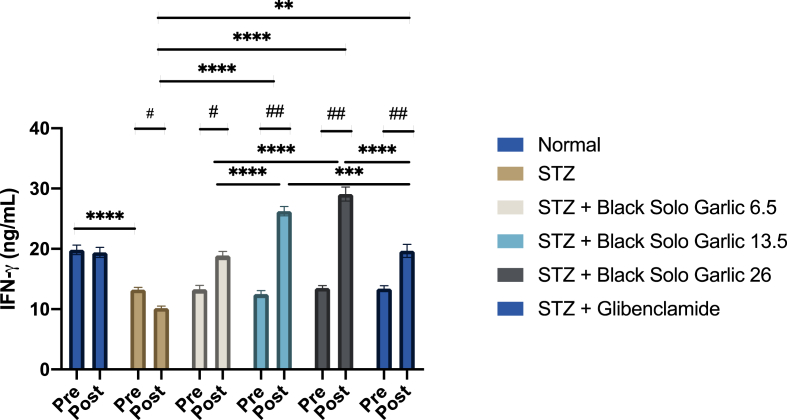
Treatment of black solo garlic increased IFN-γ. IFN-γ level in the treatment of black solo garlic in 6.5, 13.5, 26 g/kg doses and glibenclamide 0.09 mg/kg in streptozotocin-induced rats was determined by ELISA. Statistical significance for the difference among groups: ∗∗∗∗p < 0.0001; ∗∗∗p < 0.0005; ∗∗p < 0.01. Statistical significance for the difference between the data of pre-test group vs post-test groups: ^##^p < 0.01; ^#^p < 0.05.

Streptozotocin induction produced a significant decreased of IFN-γ compared to the normal group (p < 0.0001) (Figure 3). Black solo garlic (13.5 and 26 g/kg)-treated diabetic rats had significantly increased IFN-γ level compared to the untreated diabetic rats (p < 0.0001). The effects on IFN-γ were dose dependent, IFN-γ level of rats having been administrated medium or high black solo garlic dose being significantly higher than those in the diabetic rats receiving the low black solo garlic dose. Moreover, the treatment of black solo garlic at dose of 13.5 g/kg and 26 g/kg body weight was able to increase IFN-γ level higher than those of glibenclamide.

## 4 Discussion

The administration of steeping black solo garlic decreases the level of IL-1β, IL-6 and TNF-α and increases IFN-γ in experimental animals. These results suggested that black solo garlic is potential to be used as an immunomodulator and antiinflammation in diabetic condition to prevent complication.

Induction of STZ generates free radicals which oxidize β-pancreatic cells by means of alkylating DNA, damaging mitochondria, and inhibiting the O-GlcNAcase enzyme. The oxidation process induces toxicity through a free radical chain reaction. This results in an inflammatory process to organ malfunction [16], which causes the release of major pro-inflammatory cytokines such as IL-1β and TNF-α. The present study showed that the administration of steeping black solo garlic significantly reduced levels of IL-1β, IL-6 and TNF-α and increased IFN-γ in experimental animals.

Black solo garlic contains *S*-allyl-cysteine that has pharmacological effect as an antidiabetic, antioxidant, and anti-inflammatory with a higher bioactivity than regular types. Furthermore, the *S*-allyl-cysteine level with distilled water is higher than ethanol solvent [17]. Black solo garlic contains organosulphur compounds with potent antioxidant activity and free radical scavengers [18]. During inflammation, many pro-inflammatory mediators are generated and the administration of black solo garlic suppresses them to avoid tissue damage. Antioxidant compounds such as *S*-allyl cysteine, *S*-allyl mercaptocysteine, and allicin exhibit antioxidant activity and they inhibit inflammation by suppressing the activity of the NF-κB signaling [19]. Meanwhile, *S*-allyl-cysteine has been proven to scavenged superoxide anions, hydrogen peroxide, hydroxyl radicals, peroxynitric radicals, and peroxyl radicals produced in neuron, as well as hypochloric acid and singlet oxygen in microglial cells.

Chemical compounds content of solo garlic is similar to ordinary type; however, some compounds have higher amounts such as flavonoids, total phenols, *S*-allyl-cysteine, and minerals [20]. Flavonoids can function as antioxidants to reduce or terminate free radical chain reactions [21]. *S*-allyl cysteine ameliorates cells by regulating peroxisomal proliferator activator receptor-α (PPAR-α), sterol regulatory element binding protein 1c (SREBP-1c), and decreasing levels of reactive oxygen species [22]. In addition, black solo garlic contains minerals such as Cu, Mn, and Zn, which have important role in the activity of oxidant enzymes such as superoxide dismutase. The antioxidant activity reduces reactive oxygen species and increases glutathione peroxidase, catalase, superoxide dismutase, reduced glutathione (GSH) and malondialdehyde [23, 24].

Black garlic suppresses the toll-like receptor 4 (TLR4) signals in macrophages. TLR4 acts to activate myeloid differentiation factor 88 (MyD88). It releases pro-inflammatory mediators such as IL-1β, IL-6, and TNF-α [25]. These various mechanisms underlie several studies which explaining the role of black garlic in suppressing the formation of TNF-α, IL-1β, and interferon-γ [26, 27]. Although BSG affected TLR4, the effect on NLRP as a component of inflammasome that have critical role in generating IL-1β is interesting to be explored in the future study.

## Declarations

### Author contribution statement

Saryono: Conceived and designed the experiments; Performed the experiments; Analyzed and interpreted the data; Contributed reagents, materials, analysis tools or data; Wrote the paper.

Desiyani Nani: Conceived and designed the experiments; Analyzed and interpreted the data.

Atikah Proverawati: Performed the experiments; Contributed reagents, materials, analysis tools or data; Wrote the paper.

Sarmoko: Analyzed and interpreted the data; Contributed reagents, materials, analysis tools or data; Wrote the paper.

### Funding statement

This work was supported by LPPM Jenderal Soedirman University (No. T/324/UN23.18/PT.01.03/2020).

### Data availability statement

Data will be made available on request.

### Declaration of interests statement

The authors declare no conflict of interest.

### Additional information

No additional information is available for this paper.
